# Chronic Intermittent Hypobaric Hypoxia Enhances Bone Fracture Healing

**DOI:** 10.3389/fendo.2020.582670

**Published:** 2021-02-16

**Authors:** Li Zhang, Lin Jin, Jialiang Guo, Kai Bao, Jinglue Hu, Yingze Zhang, Zhiyong Hou, Liping Zhang

**Affiliations:** ^1^ Department of Orthopaedic Surgery, Third Hospital of Hebei Medical University, Shijiazhuang, China; ^2^ Department of Orthopaedic Surgery, Hebei Provincial Hospital of Traditional Chinese Medicine, Hebei University of Chinese Medicine, Shijiazhuang, China; ^3^ Department of Physiology, Hebei Medical University, Shijiazhuang, China

**Keywords:** chronic intermittent hypobaric hypoxia, bone fracture healing, hypoxia-inducible factor-1α, mechanical properties, bone mass, callus angiogenesis, bone formation

## Abstract

The effect of chronic intermittent hypobaric hypoxia (CIHH) on bone fracture healing is not elucidated. The present study aimed to investigate the role of CIHH on bone fracture healing and the mechanism. The Sprague-Dawley rats were randomly divided into the CIHH group and control group and monitored for 2, 4, or 8 weeks after femoral fracture surgery. Bone healing efficiency was significantly increased in the CIHH group as evidenced by higher high-density bone volume fractions, higher bone mineral density, higher maximum force, and higher stiffness. Histologically, the CIHH group exhibited superior bone formation, endochondral ossification, and angiogenic ability compared with the control group. The expression of HIF-1α and its downstream signaling proteins VEGF, SDF-1/CXCR4 axis were increased by the CIHH treatment. Moreover, the expression of RUNX2, osterix, and type I collagen in the callus tissues were also up-regulated in the CIHH group. In conclusion, our study demonstrated that CIHH treatment improves fracture healing, increases bone mineral density, and increases bone strength *via* the activation of HIF-1α and bone production-related genes.

## Introduction

Bone fracture is a common clinical disease, including traumatic fracture, pathologic fracture, and periprosthetic fracture. Although surgery can effectively treat the bone fracture, about 10% of fractures have impaired healing. Non-union or delayed union of fracture is still a serious public health concern (1).

Fracture healing is a complex physiological process, in which callus formation is a critical step for successful fracture healing (2). The overall stability of the fixation of the fracture also affects fracture healing, the more-extensive cartilage tissue formation, the lower stability, and more bone tissues, the higher stability (3). Moreover, angiogenesis will be increased when fractures cannot be stably fixed (4, 5). There are several strategies to enhance fracture healing clinically (6). Commonly, it can be divided into the biophysical and biological strategy. The biophysical strategy includes electromagnetic fields and low-intensity pulsed ultrasonography (6). The biological strategy includes autologous bone marrow, peptide signaling molecules, and morphogenetic factors treatment (bone morphogenetic proteins and Wnt proteins) (6). Among them, bone morphogenetic proteins (BMPs) are the most widely studied candidate for enhancing bone repair, especially BMP-2 and BMP-7. Besides, bone morphogenetic protein 2 (BMP-2) and bone morphogenetic protein 7 (BMP-7) are the only FDA-approved therapies to promote fracture healing (7). However, BMP-2 treatment did not significantly reduce the fracture healing time of fracture patients and did not substantially promote the healing of open fractures (8, 9). Compared with autologous bone transplantation, treatment with BMP-7 did not significantly improve fracture healing (6). Also, it can cause local complications as well as serious side effects (10). Therefore, safe and effective treatment after fracture remains to be developed.

The environment has been recognized to affect the fate of bone cells, including the proliferation, differentiation, mobilization of bone progenitor cells, and the activation of mature bone cells (11, 12). Chronic intermittent hypobaric hypoxia (CIHH) is a treatment with moderate hypoxia simulating high altitude interrupted by normoxia. Studies have demonstrated that CIHH has beneficial effects on multiple organs or tissues of the body, such as the heart, brain, liver, and kidney (13–15). For example, CIHH protects heart, brain, and skeletal muscle against ischemia/reperfusion injury through enhancement of antioxidation, induction of heat shock proteins, an increase of coronary flow and myocardial capillary angiogenesis, activation of adenosine triphosphate (ATP)-sensitive potassium channels, and inhibition of mitochondrial permeability transition pores (16–21). Also, CIHH protects the kidneys of diabetic rats and the livers of rats with nonalcoholic fatty liver disease (22, 23). Besides, CIHH treatment decreases the arterial blood pressure in renal vascular hypertension and metabolic syndrome rats (24–26). Besides, CIHH treatment has anti-arthritis, anti-aplastic anemia, anxiolytic- and anti-depressant like effects in mice and rats (27–29). More interesting, someone reported that CIHH promotes the healing of the drilled-hole bone defect in mice (30), or has positive effects on bone mineral density in rats (31). These shreds of evidence prompted us that CIHH may promote bone fracture healing.

In the present study, the rats were exposed to CIHH conditions or normal environment for 8 weeks post-fracture, then the radiography, micro-CT assessment, biomechanical testing, immunohistochemistry, and molecular biology techniques were employed to investigate the effect of CIHH on fracture healing and the underlying mechanism. Our data indicated that CIHH treatment accelerates fracture healing *via* the activation of HIF-1α associated signaling pathways.

## Materials and Methods

### Animals and CIHH Treatment

Adult male Sprague-Dawley rats were provided by Liaoning Changsheng Biotechnology Co., Lot. (Shenyang, Liaoning, China) with the permission number: SCXK, Liao 2015-0001. All experiments were reviewed and approved by the Ethics Committee of Hebei Medical University and carried out in compliance with the Regulatory Guideline on the Use of Experimental Animals (China, 2011).

### Fracture Model and CIHH Treatment

The femoral fracture model was established as previously reported (32). In brief, an incision was made on the lateral aspect of the right thigh after anesthesia with Chloral hydrate (350 mg/kg). After the fascia, muscle, and periosteum were separated, the midshaft femoral fracture was made with a saw, and then fixed with a 1 mm Kirshner wire. Analgesia was given by subcutaneous injections of buprenorphine (50 μg/kg of body weight) 2 hours after surgery and once a day for three consecutive days. All surgical procedures were performed under strict aseptic conditions.

The rats were randomly divided into CIHH treatment (CIHH) and control groups after bone fracture surgery. As previously described (33, 34), the rats in the CIHH group were exposed to a hypobaric hypoxia chamber (Pressure: 50 kPa, oxygen concentration: 10%-11%, 22°C ± 1°C) for 6 continuous hours (from 10:100 to 16:00) every day, and the rest time was kept in normal conditions. The control rats were housed under normal conditions. All the rats had free access to water and food.

Rats were sacrificed with over anesthesia at 2, 4, and 8 weeks post-fracture, respectively. A total of 1 cm tissues around the proximal and distal to the fracture site were collected for further analysis. For each treatment group, 6 rats were assigned to the X-radiography and micro-CT Assessment, 6 rats were assigned to the three-point bending test, 18 rats were assigned to the western blot and Real-time PCR analysis (6 in each time point), and 18 rats were assigned to the histological analysis (6 in each time point).

### X-Radiography and Micro-CT Assessment

The femoral bones were photographed by a softex X-ray apparatus (Softex CSM-2; Softex, Tokyo, Japan) at week 2, week 4, and week 8 post-fracture. Femora were scanned by a micro-CT system (Bruker-microCT, SkyScan 1176, Kontich, Antwerpen, Belgium) at 8 weeks post-fracture. The scan protocol was set as follows: 18-μm isometric voxel size, 800 mA, and 80 kVp. Taking the fracture line as the midpoint reference, a total of 200 slices between the proximal and distal were analyzed. The callus was manually drawn as a region of interest, then the bone parameters of high-density bone volume (BV), total bone volume (TV), the volume fractions (BV/TV), and bone mineral density (BMD) were analyzed with CTAn software version 1.13 (Bruker-microCT). The three-dimensional representative images were generated by CTXox software version 3.3 (Bruker-microCT).

### Three-Point Bending Test

A three-point bending test was performed using a material testing machine (Bose 3200, Eden Prairie, MN, USA). The three bearings of the load consisted of a femoral head. After checking the position of the femur was correct, the roller stamp was driven at a speed of 5 mm/min. The pressure was monitored every 0.001 mm until fracture. We were able to analyze the elasticity, maximum force, braking force, and yield strength.

### Histological Analysis

For hematoxylin-eosin (HE) staining, the bone callus samples were decalcified and embedded in paraffin. Then the samples were serially sectioned into 5-μm thickness. Next, the slices were stained with HE dye liquor. For Safranin O staining, the sections were p stained with Safranin O for 2 min (Solarbio, Beijing, China). Then the samples were treated in 95% ethanol for 3 s, treated twice in 100% ethanol for 2 min each, dewaxed twice in xylene for 10 min each. Lastly, these samples were mounted in neutral balsam (Sinopharm, Shanghai, China) and observed using a light microscope (Olympus, Tokyo, Japan).

### Immunohistochemistry (IHC)

Paraffin-embedded sections were mounted on slides, dewaxed, and dehydrated. After washing, slides were incubated with primary antibodies against platelet endothelial cell adhesion molecule 1 (PECAM-1/CD31, Affinity, Changzhou, Jiangsu, China), runt-related transcription factor (RUNX2, Affinity, Changzhou, Jiangsu, China), osterix (Affinity, Changzhou, Jiangsu, China), and type I collagen (Affinity, Changzhou, Jiangsu, China) overnight at 4°C, respectively. After washing with PBS, slides were incubated with a horseradish peroxidase-conjugated secondary antibody (Thermo Fisher, Cambridge, MA, USA) for 60 min at 37°C. Then these slides were stained with DAB (Solarbio, Beijing, China) and counterstained with hematoxylin (Solarbio, Beijing, China).

### Western Blot Analysis

The proteins from the callus tissues were extracted by using lysis buffer (Beyotime, Shanghai, China). Then the protein was quantified by the BCA assay kit (Beyotime, Shanghai, China). After that, an equal amount of proteins was electrophoresed on SDS-PAGE and then transferred onto PVDF membranes (Thermo Fisher, Cambridge, MA, USA). Next, the membranes were immuno-blotted with Hypoxia-inducible factor-1α (HIF-1α) rabbit antibody (1: 500 dilution, Catalog No.: AF1009, Affinity, Changzhou, Jiangsu, China), vascular endothelial growth factor (VEGF) rabbit antibody (1: 500 dilution, Catalog No.: bs-1313R, Bioss, Beijing, China), Runx2 rabbit antibody (1: 500 dilution, Catalog No.: AF5186, Affinity), Osterix (1: 400 dilution, Catalog No.: DF7731, Affinity), Type I collagen antibody (1: 1000 dilution, Catalog No.: AF7001, Affinity), SDF-1α rabbit antibody (1: 500 dilution, Catalog No.: AF5166, Affinity), CXCR4 antibody (1: 1000 dilution, Catalog No.: A12534, ABclonal, Wuhan, China), or β-actin mouse antibody (1: 2000 dilution, Catalog No.:60008-1-Ig, proteintech, Wuhan, Hubei, China). The membranes were washed three times and incubated with goat HRP-conjugated secondary antibodies (proteintech, Wuhan, Hubei, China). The specific bands were developed with the ECL system (7 Sea biotech, Shanghai, China). β-actin was used as the control of HIF-1α and VEGF.

### Real-Time PCR

The fracture callus was collected and the total RNA was extracted by the RNApure high purity and rapid total RNA extraction kit (Bioteke Corporation, Beijing, China). The cDNA was synthesized by the M-MLV reverse transcriptase (Takara, Beijing, China) in the presence of RNase inhibitor (Takara) as per the user’s manual. The quantitative real-time PCR reaction system includes 200 ng cDNA, forward primer, and reverse primer 0.2 μg for each, 25 μl TaKaRa Taq™ HS Perfect Mix (Takara). The PCR was carried out on Exicycler 96 amplifier (Bioneer, Daejeon, Korea). The relative mRNA level was calculated using the 2^-ΔΔCt^ method. The primers were as follows: HIF-1α (Forward: 5’-CTACTATGTCGCTTTCTTGG-3’, Reverse: 5’-GTTTCTGCTGCCTTGTATGG-3’); VEGF (Forward: 5’-CGGACAGACAGACAGACACC-3’, Reverse: 5’-AGCCCAGAAGTTGGACGAAA-3’); RUNX2 (Forward: 5’-CCATAACGGTCTTCACAAATC-3’, Reverse: 5’-GAGGCGGTCAGAGAACAAACT-3’); Osterix (Forward: 5’-AAAAGGAGGCACAAAGAAGC-3’, Reverse: 5’-GGGAAAGGGTGGGTAGTCAT-3’); COL1A1 (Forward: 5’-TCCTGCCGATGTCGCTATCC-3’, Reverse: 5’-TCGTGCAGCCATCCACAAGC-3’); SDF-1α (Forward: 5’- GCATCAGTGACGGTAAGC-3’, Reverse: 5’- GAAGGGCACAGTTTGGAG-3’); CXCR4 (Forward: 5’- GGCAATGGGTTGGTAATC-3’, Reverse: 5’- GACAATGGCAAGGTAGCG-3’), β-actin (Forward: 5’- GGAGATTACTGCCCTGGCTCCTAGC-3’, Reverse: 5’- GGCCGGACTCATCGTACTCCTGCTT-3’).

### Statistical Analysis

Data are expressed as the mean d± S.D. Statistical analyses were carried out by using Graphpad Prism 8 (GraphPad Software Inc., San Diego, CA, USA). The difference between groups was analyzed by Student’s t-test or ANOVA analysis. Differences were considered statistically significant when P < 0.05.

## Results

### Radiographic Analysis and Fracture Healing Score

**Figure 1:**
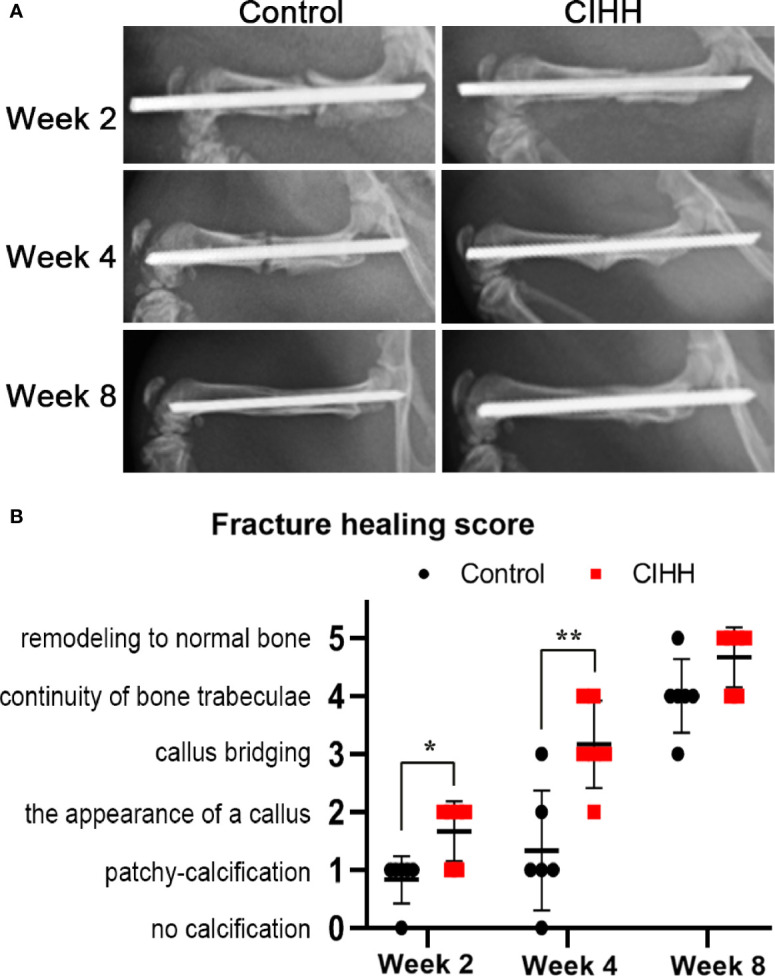
Radiographic analysis and fracture healing score. **(A)** Representative radiographs. **(B)** Fracture healing score. n=6, Mean ± SD, *p < 0.05, **p < 0.01.

Radiographic images showed that the speed of fracture healing was faster in CIHH rats than control rats at weeks 2, 4, and 8 post-fracture (**Figure 1A**). Compared with control rats, the callus amount was significantly increased in CIHH rats at week 4, and the remodeling of fracture healing was almost finished in CIHH rats. Moreover, normal bone appeared at the fracture site in the CIHH rats at week 8. The radiographic scoring of fracture healing was shown in **Figure 1B**. The average fracture healing score was 1.7 ± 0.5 in CIHH rats, significantly higher than 0.8 ± 0.4 in control rats at week 2 post-fracture (<0.05), and 3.2 ± 0.8 in CIHH rats, much higher than 1.3 ± 1.0 in control rats at week 4 (P<0.01). Although there was no statistical difference in fracture healing score between the two groups at week 8, the score was 4.6 ± 0.5 in CIHH rats, still higher than 3.8 ± 0.4 in the control group (P>0.05). The results revealed that CIHH treatment can increase callus production and accelerate bone fracture healing.

### Bone Mass and Mechanical Properties of Fracture Calluses

**Figure 2:**
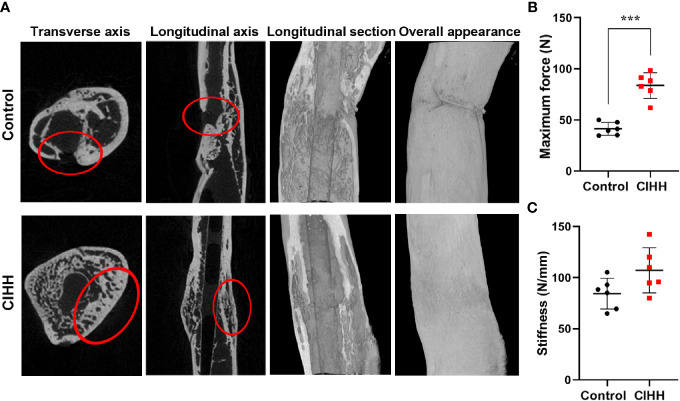
Bone mass and mechanical properties of fracture calluses. **(A)** Micro-CT images of rat femora at week 8. **(B)** Maximum force. **(C)** Stiffness. n=6, Mean ± SD, ***p < 0.001.

Micro-CT scanning showed that the fracture line was clearly visible, some bone cortex is discontinuous, and the fracture end was partially hardened in the control group. On the contrary, the CIHH group exhibited a closed fracture line and healed well (**Figure 2A**). Further, the high-density bone volume (BV), total bone volume (TV), the volume fractions (BV/TV), and bone mineral density (BMD) were also measured by micro-CT, and the results were shown in **Table 1**. The TV had no statistical difference between the CIHH rats (38.6 ± 6.7 mm^3^) and control rats (36.2 ± 3.9 mm^3^) (P>0.05). The BV was 24.9 ± 3.1 mm^3^ in CIHH rats, much higher than 18.3 ± 1.1 mm^3^ in control rats (P<0.001), and BV/TV was 65.8 ± 12.5% in CIHH rats, significantly higher than 50.1 ± 3.8% in control rats (P<0.05). Moreover, the BMD was significantly increased in CIHH rats, about 4.2% higher than control rats (P<0.05). In this study, only week 8 groups were subjected to a three-point bending test as the fractured femurs at weeks 2 and 4 were too weak to test. The maximum stress was 83.6 ± 12.6 N in CIHH rats, markedly greater than 41.4 ± 3.1 N in control rats (P<0.001, **Figure 2B**). Although there was no statistical difference of stiffness between the two groups, the stiffness was 105.9 ± 32.1 in CIHH rats, still greater than 84.3 ± 15.0 in the control group (P>0.05, **Figure 2C**). These results suggested that CIHH treatment could increase the amount of highly mineralized bone and improve fracture healing.

**Table 1:** The parameters of the bone microstructure at the fracture callus area (n=6, mean ± SD).

	TV (mm^3^)	BV (mm^3^)	BV/TV (%)	BMD (g/cm^3^)
Control	36.20 ± 3.86	18.29 ± 1.13	50.08 ± 3.82	0.95 ± 0.03
CIHH	38.59 ± 6.71	24.85 ± 3.13	65.77 ± 12.46	0.99 ± 0.01
P value	> 0.05	< 0.001	< 0.05	< 0.05

### Histological Evaluation of Fracture

**Figure 3:**
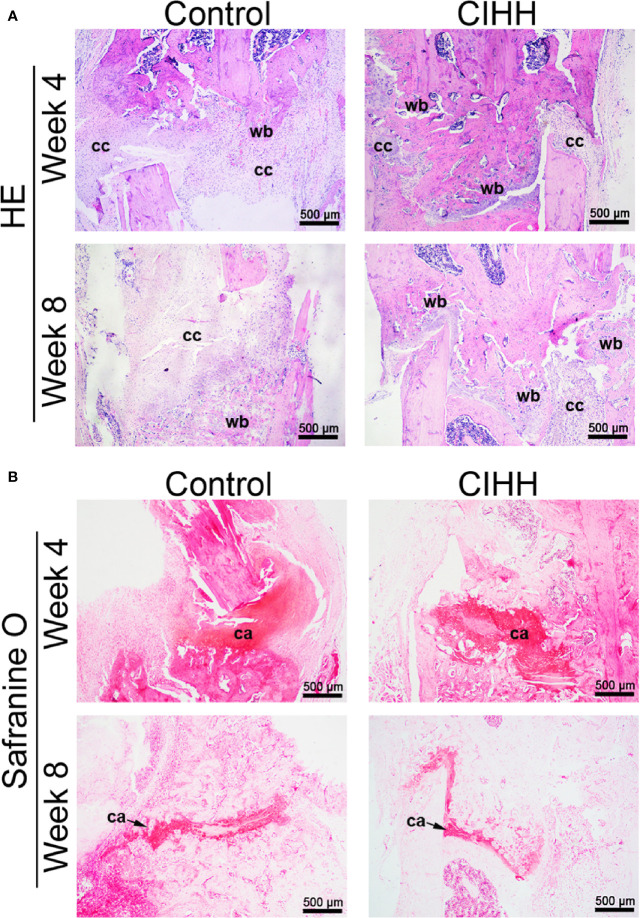
Histological evaluation of fracture. **(A)** HE staining of fracture callus sections. **(B)** Safranine O staining of fracture callus sections. Magnification, ×40; scale bar = 500 µm. The typical images from 6 repeats were shown. wb, woven bone; cc, cartilaginous callus; ca, cartilage area.

The representative histological sections of fracture sites were shown in **Figure 3**. At week 4 post-fracture, the control group exhibited the domination of callus hyperplasia along with the appearance of the woven bone, whereas a larger woven bone was developed in the CIHH group. At week 8 post-fracture, a thicker callus was formed and more woven bone was observed in control rats, while the woven bone was predominated and nearly united in the CIHH rats (**Figure 3A**). Safranin O staining showed that less cartilage tissue was observed in fracture calluses in CIHH rats compared with control rats at weeks 4 and 8 post-fracture (**Figure 3B**), indicating a better endochondral ossification process in the CIHH group.

### Angiogenesis During Fracture Healing

**Figure 4:**
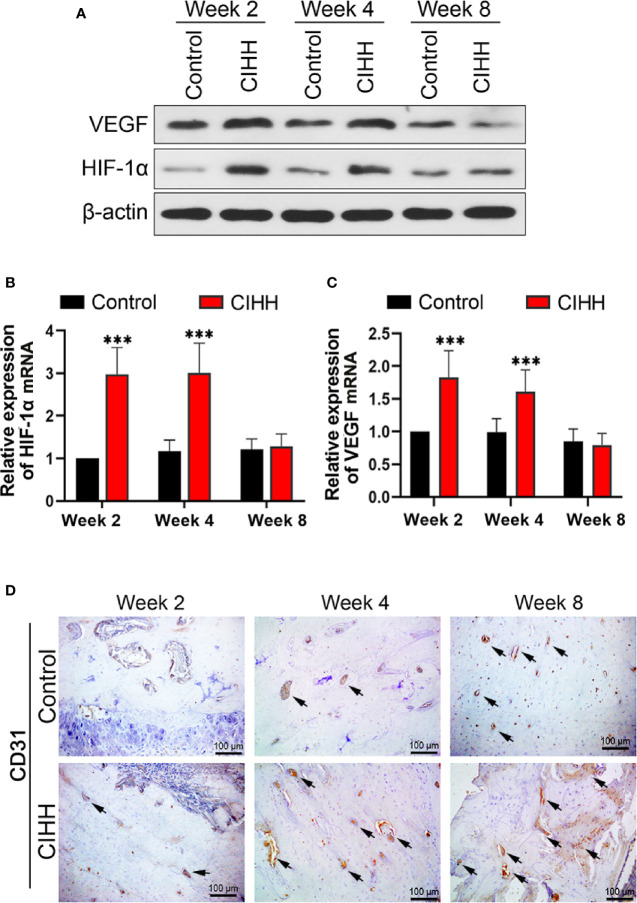
Angiogenesis. **(A)** Western blot images of HIF-1α, VEGF, and β-actin in the callus area. **(B)** Quantitative real-time PCR of the callus HIF-1α. **(C)** Quantitative real-time PCR of the callus VEGF. **(D)** Immunohistochemistry of CD31 within the woven bone. Magnification, ×200, scale bar = 100 µm. The typical CD31-positive capillaries were indicated by arrows. n=6, Mean ± SD, ***p < 0.01.

The main mechanism by which cells respond to hypoxia is through the stabilization of hypoxia-inducible factor-alpha (HIF-α) proteins. Fracture healing involves angiogenesis, and vascular endothelial growth factor (VEGF) is related to HIF-1α (35, 36). In our study, we found that the protein expression levels of HIF-1α and VEGF were enhanced in CIHH rats at week 2 and week 4 when compared to the control rats (**Figure 4A**); however, these levels were almost the same as the control rats at week 8 post-fracture. In addition, we investigated the mRNA expression levels of HIF-1α and VEGF, and the data pointed toward the same trend. HIF-1α mRNA expression in CIHH rats was about twice as high as control rats at week 2 and week 4 (P<0.001) (**Figure 4B**). VEGF mRNA expression in CIHH rats was 83.0% higher than control rats at week 2 (P<0.05) and 62.6% higher than control rats at week 4 (P<0.001) (**Figure 4C**). Moreover, to observe the blood vessels and endothelial cells around the woven bone, we performed the IHC analysis of the vascular specific marker platelet endothelial cell adhesion molecule 1 (PECAM-1; CD31). We found that the levels of CD31 were markedly increased by CIHH treatment at weeks 2, 4, and 8 (**Figure 4D**). These data suggested that CIHH treatment induced an early increase in neovascularization.

### The Expression of SDF-1 and CXCR4 Axis at Fracture Sites

**Figure 5:**
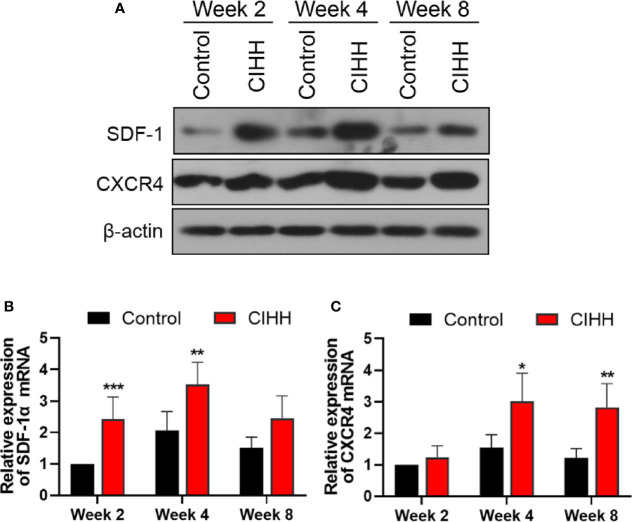
The expression of SDF-1 and CXCR4 axis at fracture sites. **(A)** Western blot images of SDF-1, CXCR4, and β-actin in the callus area. **(B)** Quantitative real-time PCR of the callus SDF-1α. **(C)** Quantitative real-time PCR of the callus CXCR4. n=6, Mean ± SD, *p < 0.05, **p < 0.01, ***p < 0.001.

The SDF-1/CXCR4 axis was reported to be regulated by the HIF-α and plays pivotal roles during progenitor homing, hematopoiesis, neovascularization, and wound healing (37). Thus, the expression of SDF-1 and CXCR4 at the fracture sites was also detected. As shown in **Figures 5A–C**, the protein and mRNA levels of SDF-1 and CXCR4 in the CIHH group were all higher in the control group at week 2, week 4, and week 8. These results demonstrated that the SDF-1/CXCR4 axis may be involved in CIHH induced fracture healing.

### Bone Formation

**Figure 6:**
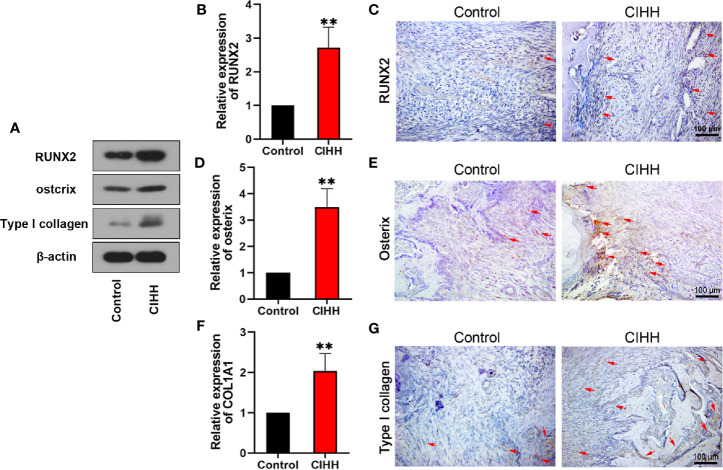
Bone formation. Protein levels of RUNX2, ostcrix, Type I collagen **(A)**. mRNA expression level of RUNX2 **(B)**, osterix **(D)**, and COL1A1 **(F)** of the callus ares. Immunohistochemistry of RUNX2 **(C)**, osterix **(E)**, and type I collagen **(G)** within the callus area. Magnification, ×200, scale bar = 100 µm. The typical positive stained cells or regions were indicated by arrows. n=6, Mean ± SD, **p < 0.01.

According to the report, active osteoblasts and collagen formation were significantly increased at week 2 post-fracture (38), so we examined the levels of related factors in the callus at week 2 post-fracture. Runt-related transcription factor (RUNX2) and osterix are biochemical markers of osteoblastic differentiation and bone formation. COL1A1 gene encodes the alpha1 chain of type I collagen. The western blot results exhibited that the protein levels of RUNX2, osterix, and type I collagen were all increased by the CIHH treatment (**Figure 6A**). The mRNA expression of RUNX2 in CIHH rats was about twice as high as that in control rats (P<0.01, **Figure 6B**), the mRNA expression of osterix in CIHH rats was about three times higher than that in control rats (P<0.01, **Figure 6D**), the mRNA expression of COL1A1 in CIHH rats was about twice as high as that in control rats (P<0.01, **Figure 6F**). These changes were further confirmed by immunohistochemical (**Figures 6C, E, G**). These findings suggested that CIHH treatment could promote osteoblastic differentiation and bone formation by the up-regulation of RUNX2, osterix, and type I collagen.

## Discussion

Fracture healing is a long and complex process, including initial inflammatory response, the formation of fibrocartilage, mineralization of callus, and bone remodeling (39). When a fracture occurs, the blood is interrupted at the fracture site, resulting in acute hypoxia and necrosis of the adjacent bone tissues (40). In the early stages of fracture healing, hypoxia could induce cartilage callus formation. HIF-1α is a master regulator of oxygen homeostasis, and its expression was induced by hypoxia (41). Reports claimed that increased expression of HIF-1α is observed in the early stage of bone regeneration (42, 43), suggesting that the expression level of HIF-1α is associated with fracture healing (40, 44). Our results revealed that CIHH treatment might promote fracture healing through up-regulating HIF-1α.

Angiogenesis is essential for fracture healing (45). VEGF, as the angiogenesis-related transcription and growth factor, is the key factor of angiogenesis (46). Significant reduction in angiogenesis and bone formation was observed in mice lacking HIF-1α, and the administration of VEGF receptor antagonists can offset the angiogenesis induced by HIF-1α (47). These suggest that VEGF is one of the downstream proteins regulated by HIF-1α (48). According to reports, mice with VEGF deficiency exhibited reduced bone mass and reduced osteoblast (49), and VEGF has a direct effect on osteoprogenitor cells to enhance fracture healing (50). Also, CD31 is concentrated at endothelial cell borders, commonly used as the marker of blood vessels and endothelium (51). As the mediator of adhesion molecules and signal transduction, CD31 can enhance blood vessel formation and cell motility (52). Our research showed that CIHH may indirectly promote fracture healing by regulating angiogenesis *via* HIF-1α/VEGF.

SDF-1 is a member of the pro-inflammatory CXC chemokine family and plays a crucial role in cell growth, development, and differentiation *via* the activation of CXCR4 (53). The expression of SDF-1 and CXCR4 was significantly up-regulated in the damaged bone surrounding tissues during fracture healing (37). The SDF-1 could recruit mesenchymal stem cells and participate in endochondral bone repair (54). Knockdown of HIF-1α repressed mesenchymal stem cell migration by the blocking of the SDF-1/CXCR4 signaling (55). In the present study, the expression of SDF-1 and CXCR4 was increased along with the HIF-1α at the callosum region of the CIHH treatment rats, indicating that CIHH may contribute to the bone formation by HIF-1α mediated activation of the SDF-1/CXCR4 signaling.

It is well known that bone development and remodeling depend on osteoblast activity. Studies have demonstrated that RUNX2 plays a key role in mammalian osteoblast development, differentiation, and bone formation (56, 57). Mice with RUNX2 deficiency showed a complete lack of bone formation (58). In addition, increased adipogenic differentiation and the loss of cartilage ossification were observed in Runx2-deficient mice (59). In contrast, overexpression of RUNX2 could promote osteogenic differentiation and enhance the rate of new bone formation (60, 61). Osterix is a transcription factor necessary for mammalian bone formation. Osterix of vertebrates is mainly expressed in osteoblasts and plays a decisive role in the later period of osteoblast differentiation (62). In osterix null mice, the cartilage develops normally, but no bone formation occurs (63). Oh et al. found that osterix is the key regulator in chondrocyte differentiation and bone growth (63). Moreover, some researchers found that osterix null mice could express RUNX2, while RUNX2/Cbfa1 null mice could not express osterix (62). Nishio et al. further proved that osterix acts downstream of RUNX2 to control the differentiation of osteoblasts (64). Therefore, RUNX2 and osterix are important osteogenic factors and regulate the development and differentiation of osteoblasts. Type I collagen is associated with bone structure and strength, accounting for 20% of the bone matrix. COL1A1 and COL1A2 are the encoding genes of type I collagen (65). Studies demonstrated that COL1A1 is linked to osteoporosis by regulating bone mineral density (66–68), and mutations in the COL1A1 gene can cause osteogenesis imperfecta (65, 69, 70). Furthermore, researchers found that osterix can induce the expression of COL1A1 (71). As expected, our data showed that CIHH treatment could increase the expression of RUNX2, osterix, and type I collagen. These suggested that CIHH could accelerate the program of osteoblastic differentiation and promote fracture healing.

In conclusion, CIHH treatment was able to enhance fracture healing, as proved by increasing bone formation, bone mass, and bone strength. Further, the mechanism by which CIHH promoted bone repair involved enhancing the expression of HIF-1α associated signaling pathways. These results support the therapeutic potential of CIHH to enhance open fracture healing.

## Data Availability Statement

The original contributions presented in the study are included in the article/supplementary materials. Further inquiries can be directed to the corresponding authors.

## Ethics Statement

The animal study was reviewed and approved by the Ethics Committee of Hebei Medical University.

## Author Contributions

LiZ conducted the experiments, wrote the manuscript, and analyzed the data. LJ, JG, and KB contributed to the experiments and data analysis. JH and YZ conducted the micro-CT and X-ray detection. ZH guided the experiments and polished the language. LipZ designed the experiments and edited the manuscript. All authors contributed to the article and approved the submitted version.

## Funding

The study was supported by the National Key R&D Program of China (Grant No. 2019YFC0120600).

## Conflict of Interest

The authors declare that the research was conducted in the absence of any commercial or financial relationships that could be construed as a potential conflict of interest.

## Abbreviations

CIHH, chronic intermittent hypobaric hypoxia; BV, high-density bone volume; TV, total bone volume; BV/TV, the volume fractions; BMD, bone mineral density; HIF-1α, Hypoxia-inducible factor-1α; VEGF, vascular endothelial growth factor; RUNX2, runt-related transcription factor.

## References

[1] Baker CE Moore-Lotridge SN Hysong AA Posey SL Robinette JP Blum DM. Bone Fracture Acute Phase Response-A Unifying Theory of Fracture Repair: Clinical and Scientific Implications. Clin Rev Bone Mineral Metab (2018) 16(4):142–58. 10.1007/s12018-018-9256-x PMC6404386 30930699

[2] Giannoudis PV Einhorn TA Marsh D. Fracture healing: The diamond concept. Injury (2007) 38:S3–6. 10.1016/S0020-1383(08)70003-2 18224731

[3] Morgan EF Salisbury Palomares KT Gleason RE Bellin DL Chien KB Unnikrishnan GU. Correlations between local strains and tissue phenotypes in an experimental model of skeletal healing. J Biomechanics (2010) 43(12):2418–24. 10.1016/j.jbiomech.2010.04.019 PMC2935472 20546756

[4] Olerud S Stromberg L. Intramedullary reaming and nailing: its early effects on cortical bone vascularization. Orthopedics (1986) 9(9):1204–8. 10.3928/0147-7447-19860901-06 3763490

[5] Wallace AL Draper ER Strachan RK McCarthy ID Hughes SP. The vascular response to fracture micromovement. Clin Orthopaedics Related Res (1994) 301):281–90. 8156689

[6] Einhorn TA Gerstenfeld LC. Fracture healing: mechanisms and interventions. Nat Rev Rheumatol (2015) 11(1):45–54. 10.1038/nrrheum.2014.164 25266456 PMC4464690

[7] Ratko TA Belinson SE Samson DJ Bonnell C Ziegler KM Aronson N. AHRQ Technology Assessments. In: . Bone Morphogenetic Protein: The State of the Evidence of On-Label and Off-Label Use. Rockville (MD: Agency for Healthcare Research and Quality (US (2010). 25855840

[8] Govender S Csimma C Genant HK Valentin-Opran A Amit Y Arbel R. Recombinant human bone morphogenetic protein-2 for treatment of open tibial fractures: a prospective, controlled, randomized study of four hundred and fifty patients. J Bone Joint Surg Am Volume (2002) 84(12):2123–34. 10.2106/00004623-200212000-00001 12473698

[9] Simmonds MC Brown JV Heirs MK Higgins JP Mannion RJ Rodgers MA. Safety and effectiveness of recombinant human bone morphogenetic protein-2 for spinal fusion: a meta-analysis of individual-participant data. Ann Internal Med (2013) 158(12):877–89. 10.7326/0003-4819-158-12-201306180-00005 23778905

[10] Wang Y Newman MR Benoit DSW. Development of controlled drug delivery systems for bone fracture-targeted therapeutic delivery: A review. Eur J Pharmaceutics Biopharm Off J Arbeitsgemeinschaft fur Pharmazeutische Verfahrenstechnik Evol (2018) 127:223–36. 10.1016/j.ejpb.2018.02.023 PMC5948156 29471078

[11] Tian Y Ma X Yang C Su P Yin C Qian AR. The Impact of Oxidative Stress on the Bone System in Response to the Space Special Environment. Int J Mol Sci (2017) 18(10):2132. 10.3390/ijms18102132 PMC5666814 29023398

[12] Cordonnier T Layrolle P Gaillard J Langonne A Sensebe L Rosset P. 3D environment on human mesenchymal stem cells differentiation for bone tissue engineering. J Mater Sci Mater Med (2010) 21(3):981–7. 10.1007/s10856-009-3916-9 19856200

[13] Zhang Y Zhou ZN. [Beneficial effects of intermittent hypobaric hypoxia on the body]. Zhongguo ying yong sheng li xue za zhi = Zhongguo yingyong shenglixue zazhi = Chin J Appl Physiol (2012) 28(6):504–9. 23581179

[14] Zhang S Guo Z Yang S Ma H Fu C Wang S. Chronic intermittent hybobaric hypoxia protects against cerebral ischemia via modulation of mitoKATP. Neurosci Lett (2016) 635:8–16. 10.1016/j.neulet.2016.10.025 27760384

[15] Tian YM Guan Y Tian SY Yuan F Zhang L Zhang Y. Short-term Chronic Intermittent Hypobaric Hypoxia Alters Gut Microbiota Composition in Rats. Biomed Environ Sci BES (2018) 31(12):898–901. 10.3967/bes2018.122 30636661

[16] Cheng WJ Liu X Zhang L Guo XQ Wang FW Zhang Y. Chronic intermittent hypobaric hypoxia attenuates skeletal muscle ischemia-reperfusion injury in mice. Life Sci (2019) 231:116533. 10.1016/j.lfs.2019.06.008 31173783

[17] Zhang Y Zhong N Zhou ZN. Estradiol potentiates antiarrhythmic and antioxidative effects of intermittent hypoxic rat heart. Acta pharmacologica Sin (2000) 21(7):609–12. 11360667

[18] Zhong N Zhang Y Zhu HF Wang JC Fang QZ Zhou ZN. Myocardial capillary angiogenesis and coronary flow in ischemia tolerance rat by adaptation to intermittent high altitude hypoxia. Acta pharmacologica Sin (2002) 23(4):305–10. 11931703

[19] Zhong N Zhang Y Fang QZ Zhou ZN. Intermittent hypoxia exposure-induced heat-shock protein 70 expression increases resistance of rat heart to ischemic injury. Acta pharmacologica Sin (2000) 21(5):467–72. 11324449

[20] Zhu HF Dong JW Zhu WZ Ding HL Zhou ZN. ATP-dependent potassium channels involved in the cardiac protection induced by intermittent hypoxia against ischemia/reperfusion injury. Life Sci (2003) 73(10):1275–87. 10.1016/s0024-3205(03)00429-6 12850243

[21] Zhu WZ Xie Y Chen L Yang HT Zhou ZN. Intermittent high altitude hypoxia inhibits opening of mitochondrial permeability transition pores against reperfusion injury. J Mol Cell Cardiol (2006) 40(1):96–106. 10.1016/j.yjmcc.2005.09.016 16288778

[22] Tian YM Guan Y Li N Ma HJ Zhang L Wang S. Chronic intermittent hypobaric hypoxia ameliorates diabetic nephropathy through enhancing HIF1 signaling in rats. Diabetes Res Clin Pract (2016) 118:90–7. 10.1016/j.diabres.2016.06.021 27351799

[23] Yuan F Teng X Guo Z Zhou JJ Zhang Y Wang S. Chronic intermittent hypobaric hypoxia ameliorates endoplasmic reticulum stress mediated liver damage induced by fructose in rats. Life Sci (2015) 121:40–5. 10.1016/j.lfs.2014.11.019 25476828

[24] Guan Y Li N Tian YM Zhang L Ma HJ Maslov LN. Chronic intermittent hypobaric hypoxia antagonizes renal vascular hypertension by enhancement of vasorelaxation via activating BKCa. Life Sci (2016) 157:74–81. 10.1016/j.lfs.2016.05.028 27216772

[25] Zhou JJ Wei Y Zhang L Zhang J Guo LY Gao C. Chronic intermittent hypobaric hypoxia prevents cardiac dysfunction through enhancing antioxidation in fructose-fed rats. Can J Physiol Pharmacol (2013) 91(5):332–7. 10.1139/cjpp-2012-0059 23656204

[26] Li N Guan Y Tian YM Ma HJ Zhang X Zhang Y. Chronic Intermittent Hypobaric Hypoxia Ameliorates Renal Vascular Hypertension Through Up-regulating NOS in Nucleus Tractus Solitarii. Neurosci Bull (2019) 35(1):79–90. 10.1007/s12264-018-00330-z 30617765 PMC6357268

[27] Shi M Cui F Liu AJ Ma HJ Cheng M Song SX. The protective effects of chronic intermittent hypobaric hypoxia pretreatment against collagen-induced arthritis in rats. J Inflammation (London England) (2015) 12:23. 10.1186/s12950-015-0068-1 PMC4389442 25861246

[28] Yang J Zhang L Wang H Guo Z Liu Y Zhang Y. Protective Effects of Chronic Intermittent Hypobaric Hypoxia Pretreatment against Aplastic Anemia through Improving the Adhesiveness and Stress of Mesenchymal Stem Cells in Rats. Stem Cells Int (2017) 2017:5706193. 10.1155/2017/5706193 28798776 PMC5534323

[29] Zhu XH Yan HC Zhang J Qu HD Qiu XS Chen L. Intermittent hypoxia promotes hippocampal neurogenesis and produces antidepressant-like effects in adult rats. J Neurosci Off J Soc Neurosci (2010) 30(38):12653–63. 10.1523/jneurosci.6414-09.2010 PMC6633584 20861371

[30] Durand M Collombet JM Frasca S Begot L Lataillade JJ Le Bousse-Kerdilès MC. In vivo hypobaric hypoxia performed during the remodeling process accelerates bone healing in mice. Stem Cells Trans Med (2014) 3(8):958–68. 10.5966/sctm.2013-0209 PMC4116247 24944208

[31] Guner I Uzun DD Yaman MO Genc H Gelisgen R Korkmaz GG. The effect of chronic long-term intermittent hypobaric hypoxia on bone mineral density in rats: role of nitric oxide. Biol Trace element Res (2013) 154(2):262–7. 10.1007/s12011-013-9722-8 23771686

[32] Suen PK He YX Chow DH Huang L Li C Ke HZ. Sclerostin monoclonal antibody enhanced bone fracture healing in an open osteotomy model in rats. J Orthop Res (2014) 32(8):997–1005. 10.1002/jor.22636 24782158

[33] Cui F Guan Y Guo J Tian YM Hu HF Zhang XJ. Chronic intermittent hypobaric hypoxia protects vascular endothelium by ameliorating autophagy in metabolic syndrome rats. Life Sci (2018) 205:145–54. 10.1016/j.lfs.2018.05.008 29733850

[34] Yuan F Zhang L Li YQ Teng X Tian SY Wang XR. Chronic Intermittent Hypobaric Hypoxia Improves Cardiac Function through Inhibition of Endoplasmic Reticulum Stress. Sci Rep (2017) 7(1):7922. 10.1038/s41598-017-08388-x 28801645 PMC5554163

[35] Li Y Liu Y Wang C Xia WR Zheng JY Yang J. Succinate induces synovial angiogenesis in rheumatoid arthritis through metabolic remodeling and HIF-1alpha/VEGF axis. Free Radical Biol Med (2018) 126:1–14. 10.1016/j.freeradbiomed.2018.07.009 30030103

[36] Zhi Z Yang W Liu L Jiang X Pang L. Early missed abortion is associated with villous angiogenesis via the HIF-1alpha/VEGF signaling pathway. Arch Gynecol Obstet (2018) 298(3):537–43. 10.1007/s00404-018-4802-9 PMC6096576 29951709

[37] Arakura M Lee SY Takahara S Okumachi E Iwakura T Fukui T. Altered expression of SDF-1 and CXCR4 during fracture healing in diabetes mellitus. Int Orthop (2017) 41(6):1211–7. 10.1007/s00264-017-3472-8 28412763

[38] Kon T Cho TJ Aizawa T Yamazaki M Nooh N Graves D. Expression of osteoprotegerin, receptor activator of NF-kappaB ligand (osteoprotegerin ligand) and related proinflammatory cytokines during fracture healing. J Bone Miner Res (2001) 16(6):1004–14. 10.1359/jbmr.2001.16.6.1004 11393777

[39] Loi F Cordova LA Pajarinen J Lin TH Yao Z Goodman SB. Inflammation, fracture and bone repair. Bone (2016) 86:119–30. 10.1016/j.bone.2016.02.020 PMC4833637 26946132

[40] Li W Wang K Liu Z Ding W. HIF-1alpha change in serum and callus during fracture healing in ovariectomized mice. Int J Clin Exp Pathol (2015) 8(1):117–26. PMC4348821 25755698

[41] Masoud GN Li W. HIF-1alpha pathway: role, regulation and intervention for cancer therapy. Acta Pharm Sin B (2015) 5(5):378–89. 10.1016/j.apsb.2015.05.007 PMC4629436 26579469

[42] Huang J Liu L Feng M An S Zhou M Li Z. Effect of CoCl(2) on fracture repair in a rat model of bone fracture. Mol Med Rep (2015) 12(4):5951–6. 10.3892/mmr.2015.4122 26239779

[43] Komatsu DE Hadjiargyrou M. Activation of the transcription factor HIF-1 and its target genes, VEGF, HO-1, iNOS, during fracture repair. Bone (2004) 34(4):680–8. 10.1016/j.bone.2003.12.024 15050899

[44] Qiao J Huang J Zhou M Cao G Shen H. Inhibition of HIF-1alpha restrains fracture healing via regulation of autophagy in a rat model. Exp Ther Med (2019) 17(3):1884–90. 10.3892/etm.2018.7115 PMC6364185 30783464

[45] Colnot C Thompson Z Miclau T Werb Z Helms JA. Altered fracture repair in the absence of MMP9. Development (2003) 130(17):4123–33. 10.1242/dev.00559 PMC2778064 12874132

[46] Mayr-Wohlfart U Waltenberger J Hausser H Kessler S Gunther KP Dehio C. Vascular endothelial growth factor stimulates chemotactic migration of primary human osteoblasts. Bone (2002) 30(3):472–7. 10.1016/s8756-3282(01)00690-1 11882460

[47] Fielden ED. Artificial insemination in the dog. N Z Vet J (1971) 19(8):178–84. 10.1080/00480169.1971.33962 4943525

[48] Lee LT Wong YK Chan MY Chang KW Chen SC Chang CT. The correlation between HIF-1 alpha and VEGF in oral squamous cell carcinomas: Expression patterns and quantitative immunohistochemical analysis. J Chin Med Assoc (2018) 81(4):370–5. 10.1016/j.jcma.2017.06.025 29289482

[49] Liu Y Berendsen AD Jia S Lotinun S Baron R Ferrara N. Intracellular VEGF regulates the balance between osteoblast and adipocyte differentiation. J Clin Invest (2012) 122(9):3101–13. 10.1172/jci61209 PMC3428080 22886301

[50] Keramaris NC Calori GM Nikolaou VS Schemitsch EH Giannoudis PV. Fracture vascularity and bone healing: a systematic review of the role of VEGF. Injury (2008) 39(Suppl 2):S45–57. 10.1016/s0020-1383(08)70015-9 18804573

[51] Newman PJ Newman DK. Signal transduction pathways mediated by PECAM-1: new roles for an old molecule in platelet and vascular cell biology. Arterioscler Thromb Vasc Biol (2003) 23(6):953–64. 10.1161/01.atv.0000071347.69358.d9 12689916

[52] Cao G O’Brien CD Zhou Z Sanders SM Greenbaum JN Makrigiannakis A. Involvement of human PECAM-1 in angiogenesis and in vitro endothelial cell migration. Am J Physiol Cell Physiol (2002) 282(5):C1181–90. 10.1152/ajpcell.00524.2001 11940533

[53] Dar A Goichberg P Shinder V Kalinkovich A Kollet O Netzer N. Chemokine receptor CXCR4-dependent internalization and resecretion of functional chemokine SDF-1 by bone marrow endothelial and stromal cells. Nat Immunol (2005) 6(10):1038–46. 10.1038/ni1251 16170318

[54] Liu X Zhou C Li Y Ji Y Xu G Wang X. SDF-1 promotes endochondral bone repair during fracture healing at the traumatic brain injury condition. PloS One (2013) 8(1):e54077. 10.1371/journal.pone.0054077 23349789 PMC3551938

[55] Xue Y Li Z Wang Y Zhu X Hu R Xu W. Role of the HIF1alpha/SDF1/CXCR4 signaling axis in accelerated fracture healing after craniocerebral injury. Mol Med Rep (2020) 22(4):2767–74. 10.3892/mmr.2020.11361 PMC7453606 32945380

[56] Komori T. Molecular Mechanism of Runx2-Dependent Bone Development. Mol Cells (2020) 43(2):168–75. 10.14348/molcells.2019.0244 PMC7057844 31896233

[57] Kim HJ Kim WJ Ryoo HM. Post-Translational Regulations of Transcriptional Activity of RUNX2. Mol Cells (2019) 43(2):160–7. 10.14348/molcells.2019.0247 PMC7057842 31878768

[58] Komori T Yagi H Nomura S Yamaguchi A Sasaki K Deguchi K. Targeted disruption of Cbfa1 results in a complete lack of bone formation owing to maturational arrest of osteoblasts. Cell (1997) 89(5):755–64. 10.1016/s0092-8674(00)80258-5 9182763

[59] Enomoto H Furuichi T Zanma A Yamana K Yoshida C Sumitani S. Runx2 deficiency in chondrocytes causes adipogenic changes in vitro. J Cell Sci (2004) 117(Pt 3):417–25. 10.1242/jcs.00866 14702386

[60] Phillips JE Guldberg RE Garcia AJ. Dermal fibroblasts genetically modified to express Runx2/Cbfa1 as a mineralizing cell source for bone tissue engineering. Tissue Eng (2007) 13(8):2029–40. 10.1089/ten.2006.0041 17516856

[61] Zhang X Yang M Lin L Chen P Ma KT Zhou CY. Runx2 overexpression enhances osteoblastic differentiation and mineralization in adipose–derived stem cells in vitro and in vivo. Calcif Tissue Int (2006) 79(3):169–78. 10.1007/s00223-006-0083-6 16969589

[62] Nakashima K Zhou X Kunkel G Zhang Z Deng JM Behringer RR. The novel zinc finger-containing transcription factor osterix is required for osteoblast differentiation and bone formation. Cell (2002) 108(1):17–29. 10.1016/s0092-8674(01)00622-5 11792318

[63] Oh JH Park SY de Crombrugghe B Kim JE. Chondrocyte-specific ablation of Osterix leads to impaired endochondral ossification. Biochem Biophys Res Commun (2012) 418(4):634–40. 10.1016/j.bbrc.2012.01.064 PMC4012832 22290230

[64] Nishio Y Dong Y Paris M O’Keefe RJ Schwarz EM Drissi H. Runx2-mediated regulation of the zinc finger Osterix/Sp7 gene. Gene (2006) 372:62–70. 10.1016/j.gene.2005.12.022 16574347

[65] Augusciak-Duma A Witecka J Sieron AL Janeczko M Pietrzyk JJ Ochman K. Mutations in the COL1A1 and COL1A2 genes associated with osteogenesis imperfecta (OI) types I or III. Acta Biochim Pol (2018) 65(1):79–86. 10.18388/abp.2017_1612 29543922

[66] Stewart TL Jin H McGuigan FE Albagha OM Garcia-Giralt N Bassiti A. Haplotypes defined by promoter and intron 1 polymorphisms of the COLIA1 gene regulate bone mineral density in women. J Clin Endocrinol Metab (2006) 91(9):3575–83. 10.1210/jc.2005-2651 16804049

[67] Mann V Hobson EE Li B Stewart TL Grant SF Robins SP. A COL1A1 Sp1 binding site polymorphism predisposes to osteoporotic fracture by affecting bone density and quality. J Clin Invest (2001) 107(7):899–907. 10.1172/jci10347 11285309 PMC199568

[68] Stover DA Verrelli BC. Comparative vertebrate evolutionary analyses of type I collagen: potential of COL1a1 gene structure and intron variation for common bone-related diseases. Mol Biol Evol (2011) 28(1):533–42. 10.1093/molbev/msq221 20724381

[69] Tanner L Vainio P Sandell M Laine J. Novel COL1A1 Mutation c.3290G>T Associated With Severe Form of Osteogenesis Imperfecta in a Fetus. Pediatr Dev Pathol (2017) 20(5):455–9. 10.1177/1093526616686903 28812463

[70] Symoens S Steyaert W Demuynck L De Paepe A Diderich KE Malfait F. Tissue-specific mosaicism for a lethal osteogenesis imperfecta COL1A1 mutation causes mild OI/EDS overlap syndrome. Am J Med Genet A (2017) 173(4):1047–50. 10.1002/ajmg.a.38135 28261977

[71] Ortuno MJ Susperregui AR Artigas N Rosa JL Ventura F. Osterix induces Col1a1 gene expression through binding to Sp1 sites in the bone enhancer and proximal promoter regions. Bone (2013) 52(2):548–56. 10.1016/j.bone.2012.11.007 23159876

